# Address, Read before the Brooklyn Dental Association

**Published:** 1865-12

**Authors:** G. A. Mills


### ADDRESS.

BY G. A. MILLS.

Read before the Brooklyn Dental Association, Oct. 18, 1865.

Mr. President and Gentlemen of this Association :—We have before us this evening a subject that should deeply interest the brightest and dullest of our number, some, are *well* prepared to speak upon it, others, may not be—(or think so at least,) but is there one of us that can say nothing?

"Open thy mouth and I will fill it," saith the divine promise.

For what is the mouth given us? Is it for the sole purpose of receiving nutriment for the body? The Scripture says, "out of the abundance of the heart, the mouth speaketh," and "perfect love casteth out fear." Gentlemen, do we have in our hearts that love for a daily dental education, that will stimulate us to seek this knowledge, which to many of us is hidden? We *need* to know much, to practice our specialty legitimately, for I claim that we have *no right* to the title of Dentist, if we are unwilling to put forth our best energies to understand all that is closely allied to our daily practice. We *should have*, high and holy aspirations, to place ourselves before our patients with such preparation, that we may vindicate the claims they rightly have upon us. We are *not* as Webster has termed us, "tooth tinkers," if we improve the opportunities that are given us.

These social meetings *may*, and will, I trust, be of lasting advantage, in assisting all who enjoy them, in their daily researches. To say we cannot learn much, would be folly, after the experience we have had in the last three years. *Many mouths* have spoken, that have been before accustomed to be dumb. Out of the *abundance* of their hearts, their mouths *have spoken.*

We should *aim* to be useful in the calling we have chosen, that we can at the close of our professional life, look back upon one well spent.

We are advancing into a new era of duty, and it well becomes us, to faithfully survey the path that is before us. The handwriting of the past is against us, it is on the wall — and it should stimulate us for the future. Repentance brings forgiveness.

Such a position can be attained by the Dental profession, that we can throw upon the horizon of the past a halo of light, that will cover from our sight the multitude of sins, that have been committed, and *alone by wilful ignorance.*

New responsibilities have of late attached themselves to us, by the hearty recognition of the medical profession, at Chicago, and it becomes us to act well our part, "as we *sow*, so *shall* we reap." We have not created our vessels unto honor, but unto dishonor. I do not think the world waits long to attach praise to *whom* it rightfully belongs. The world *does* more, although the vision of some may wilfully fail to discern the fact. So let our motto be ever, *progress.*

He that will not adopt such a motto, let him *memento mori*, for virtually his professional life is at an end.

We are to-night called to discuss the Anatomy and Physiology of the mouth, and in so doing, doubtless, old ideas may be put forth, and may we not hope new ones? Wise men before us, have not in their day fathomed *all* that is to be known of the structure of the human body. We should not be satisfied with what we can see with the naked vision, but call to our aid the instruments of science, that we may refresh ourselves in what was once hidden mysteries.

To some extent speculations are safe.

I can but admire the man who has, by his keener intelligence made discoveries, which may not be discernable to another of *less* intelligence, although he may be *earnest*; yet *dares*

to announce them, subject, as he will be often, to the aspersions of his scientific friends We are told by *some*, that it is well to be somewhat cautious in the announcement of a new idea, or discovery, for fear that we may reflect discredit to some extent, upon those that have been looked upon as knowing all that was to be known. Some may claim that this is not the time or place, to introduce speculative ideas. I answer, *now* is the only *time* that is promised; so I say, let all speak that which is in their hearts to speak.

Anatomy, we read, is properly called the science of all organization, though commonly limited to the human body; also, meaning to cut, to disect, so as to expose the structure, situation, and use of the various parts. We have comparative anatomy, the study of all the organs of animals generally; descriptive, the anatomy of the various organs of the human body, including their shapes, mutual relations, &c.; anatomy general, treating of structure and properties of the different tissues common to several organs, embracing an examination of the general character of *all* the organs; anatomy pathological, or morbid, treating of diseased states, or alteration of structure; anatomy special, treating of the healthy state of organs; anatomy transcendental, meaning the investigation of the plan or model, upon which the living frame, and its organs are formed.

Physiology is a science which treats of the laws of life and the functions of the living being. It is divided into human and comparative; the former referring to man, the latter to animals and vegetables. Also, divided into general and special, one relating to the general laws of life, the other to the functions of individual organs.

The mouth consists of many complications of mechanism, making a very essential part of the human body, with large range of sympathy, a great variety of organs, and performing many different functions. It consists of bone, ligaments, muscle, gland, vessel, nerve, cellular, adipose tissue, mucous membrane, &c. Could we have met with man before his fall, we could have seen him in a state of perfection, with no trait of disease, caused alone by *sin*, disfiguring all that was as beautiful as the stamp of his great creator could make him. Could man return to his first estate, there would be no need of the Dentist. Those beautiful organs the teeth, the cause of so much suffering and complaint, would be free from all the imperfections with which we find them so deeply complicated. It is a sad condition of things, with the dental profession as a mass, that they assume to know so much, and yet, accomplish so little. It would seem to be true, that he who reads least, knows most. But the truth is, he who studies *most*, feels that he knows *little*. The greatest amount of knowledge we can secure in this life is faint, in consideration to that which is to be known.

How few men feel that wisdom is better than riches. When a man thinketh himself to be something — then he is in great danger of doing much mischief, and ignorance becomes to him bliss; and he quietly locks himself up in his own sphere, and rests satisfied; until he *feels* and *sees* that the irresistable tide of progression is sweeping past him, bearing away upon its bosom, that which his own egotism and self sufficiency have led him to feel secure in.

For instance, he may be called upon to diagnose a case of alveolar abscess. He will then assume to be wise, and tell his patient to be *free* from further trouble, *extraction* is the *only* radical cure; or, to quiet his conscience, (if there is necessity for it) he says, " tolerate it for s short time, and it will cure itself." Now, a conflict of idea takes place between patient and operator; he (the patient) has learned to put a higher value upon his teeth, this he has been encouraged to do, by the dentist he has employed with so much confidence, and cites similar cases of his friend, or friends, that have been radically cured by a course of treatment in the hands of A. B. and C, and rather than submit to the loss of so valuable an organ to the human body, the patient prefers to submit the case to one with whom progression has gained the ascendancy over old ideas. If asked why teeth decay, a multltude of reasons are given in a very unintelligent manner. So in fact no one is the wiser. Many say, to fill the brain so full of theories, and technical phrases, that it will befog all the practical ideas he may have. Theory and practice, should always be as far as possible, closely allied. How many of us understand, or even recognize, that the greatest attribute of Gods' character — *which is love* — enters into all the laws of nature? And it is only by this love, that the different elements which make up our bodies are allowed to remain together at all, and it is often remarked that it is a wonder that we exist as long as we do — there is so much in us that is unharmonious — keeping up a constant struggle — crying "peace when there is no peace." The law of love must predominate, — could it be dethroned, all creation would be in a chaotic state, and we would have no existence. We must recognize in our practice, this law of love, and affinity, if we would be successful in our pathological, surgical and mechanical operations, for it is the key that unlocks the casket of success. The more we recognize the fact, the more speedy will be our progress.

